# Iris Color Matters—A Contractility Analysis With Dynamic Volume-Rendered Optical Coherence Tomography Pupillometry

**DOI:** 10.1167/tvst.11.11.6

**Published:** 2022-11-07

**Authors:** Philippe Valmaggia, Nadja Inglin, Pascal Kaiser, Hendrik P. N. Scholl, Peter M. Maloca

**Affiliations:** 1 Institute of Molecular and Clinical Ophthalmology Basel, Basel, Switzerland; 2 Department of Ophthalmology, University Hospital Basel, Basel, Switzerland; 3 Supercomputing Systems AG, Zürich, Switzerland; 4 Moorfields Eye Hospital NHS Foundation Trust, London, UK

**Keywords:** iris color, pupillometry, pupillary ejection fraction, optical coherence tomography

## Abstract

**Purpose:**

To analyze natural variability in pupillary contractility with dynamic volume-rendered optical coherence tomography (OCT) pupillometry regarding iris color, age, and sex in healthy Caucasian participants.

**Methods:**

The intrapupillary spaces (IPSs) derived from anterior segment swept-source OCT of 71 healthy eyes were retrospectively analyzed. Baseline scotopic and photopic volumes and the functional parameters of pupillary ejection fraction (PEF), three-dimensional (3D) contractility, and relative light response (RLR) were measured on the swept-source OCT volumes. The effect on these parameters of iris color (brown, green, and blue), age, and sex was assessed.

**Results:**

More pigmented irises were more contractile than less pigmented irises. Iris color significantly affected scotopic baseline IPSs (brown, 10.39 ± 4.86 mm^3^; green, 9.68 ± 3.31 mm^3^; blue, 6.75 ± 4.27 mm^3^; *P* = 0.018), PEF (brown, 90.8% ± 2.7%; green, 89.1% ± 2.5%; blue, 85.0% ± 9.3%; *P* = 0.010), 3D contractility (brown, 9.52 ± 4.59 mm^3^; green, 8.66 ± 3.07 mm^3^; blue, 6.44 ± 4.87 mm^3^; *P* = 0.016), and RLR (brown, 11.90 ± 4.03; green, 9.75 ± 2.73; blue, 8.52 ± 3.88; *P* = 0.026). Absolute scotopic volume (*P* = 0.022) and 3D contractility (*P* = 0.024) decreased with age. Sex showed no correlations.

**Conclusions:**

The natural variability of pupillary contractility can be analyzed with dynamic OCT pupillometry. Iris color and age can impact pupillary response with this method.

**Translational Relevance:**

Iris contractility parameters can be measured using a commercially available OCT system, allowing for quantification of the aqueous humor volume inside the pupil.

## Introduction

Optical coherence tomography (OCT) has created unprecedented imaging possibilities in ophthalmology by enabling visualization of the ocular structures in three dimensions on a micrometer scale.^1^ Many novel applications have been developed since the invention of OCT, and today the technique is widely adopted in ophthalmic clinics.^2^ After initial imaging of the posterior pole, OCT imaging was adapted for the anterior segment, enabling visualization of the cornea, sclera, anterior chamber angle, and iris.^1^^–^^3^ Recently, three-dimensional (3D) rendering of the pupil has been made possible with a commercially available OCT device.^4^

The main function of the pupil is to serve as an aperture. The iris constrictor and the dilator muscles adjust the size of the pupil when stimulated by either the parasympathetic or sympathetic nervous system. The aperture of the pupil regulates the amount of incident light, protecting the retina from too much illumination and allowing it to adapt to scotopic conditions by increasing incident light. Thus, it can optimize the visual acuity for a wide spectrum of luminances.^5^ Besides being a regulator of light, the pupil is also a gate for the aqueous humor circulating from the ciliary body to the anterior chamber, where it is drained from the trabecular meshwork toward the draining veins. The function of the pupils can be regarded as a water pump system that pushes parts of the aqueous humor via contraction further toward the anterior chamber, where the flow through the aperture is regulated.^4^

Most evaluations of pupillary function are based on the use of pen torches, infrared dynamic pupillometry, or smartphone-based methods.^6^^–^^8^ Pupillary light response is a well-established parameter for assessing neurological afferent and efferent pathways of the eye. However, the reproducibility of such measurements is often poor, both in subjective assessments and for quantitative approaches.^9^^–^^12^ The maximum pupillary contractility can be decreased in diseases such as diabetes, optic neuritis, or non-arteritic anterior ischemic optic neuropathy or due to physiological factors such as aging.^13^^–^^16^ Another prominent physiological parameter in direct proximity to the pupil is the iris color; however, few studies have investigated its correlation with pupillary contractility, and their results on the effect of iris color have been inconclusive.^17^^–^^19^ There have been few studies on the natural variability in pupillary contractility, specifically regarding the impact of iris color and sex, and none has used volumetric OCT technologies.^19^

Therefore, in this study, we analyzed the natural variability in pupillary contractility derived from a recently reported volumetric OCT acquisition method in a healthy Caucasian subgroup.^4^ Because the changes to the aqueous humor inside the pupil cannot be accurately evaluated using planar imaging, a volumetric analysis was required. The pupillary contractility was measured with dynamic OCT pupillometry providing 3D intrapupillary space (IPS) volumes and was evaluated regarding the main factors of iris color, age, and sex.

## Methods

### Study Participants

**Figure 1.**
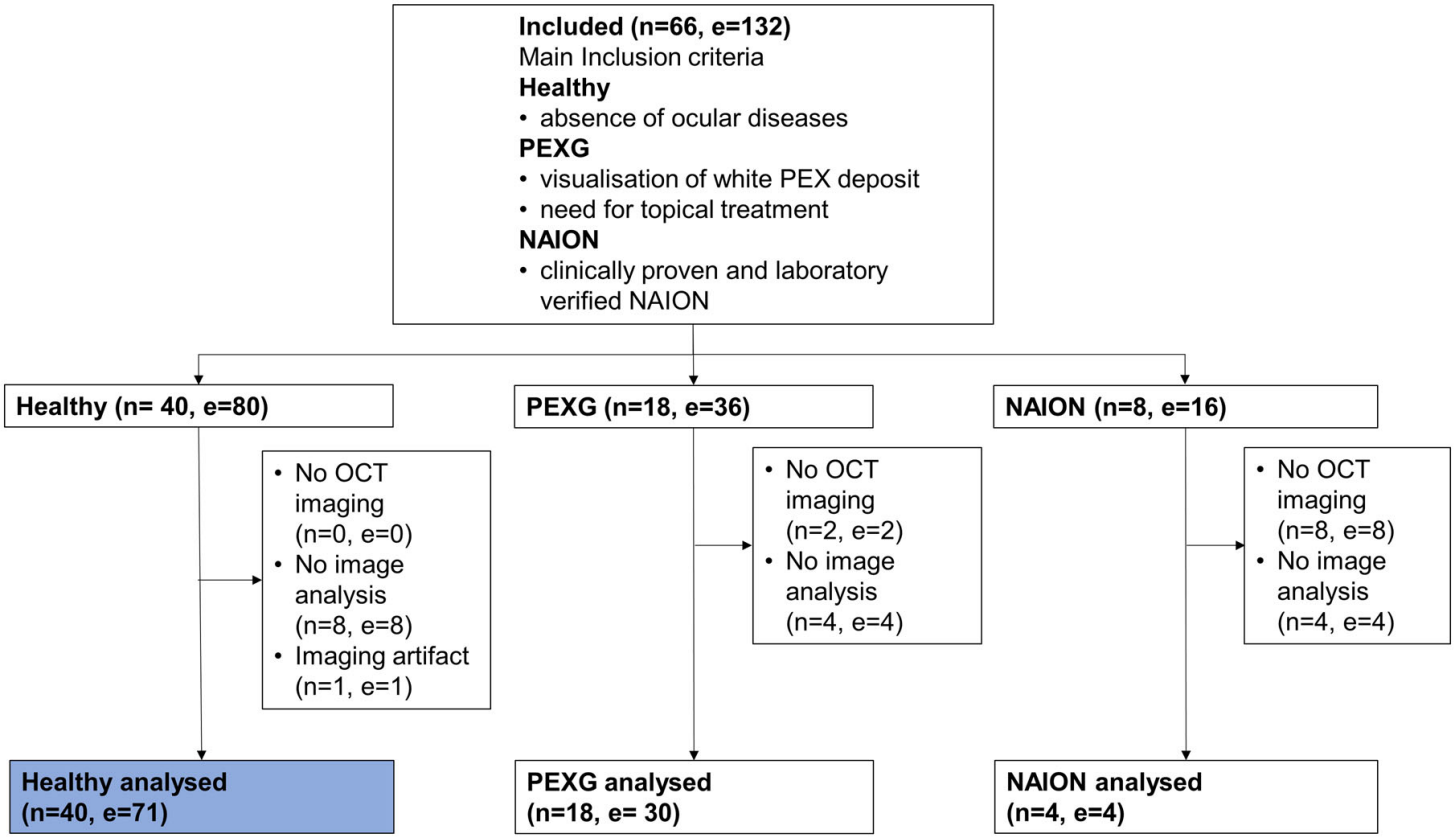
Study flowchart including the umbrella study population. The present study investigated the subgroup of healthy participants, highlighted in *blue*. PEXG, pseudoexfoliation glaucoma; NAION, non-arteritic anterior ischemic optic neuropathy; *n*, number of participants; *e*, number of eyes.

This retrospective study analyzed a subgroup of participants presented in an earlier publication.^4^ In this previous study, a method for dynamic volume-rendered OCT pupillometry was presented by comparing participants with pseudoexfoliation glaucoma or non-arteritic anterior ischemic optic neuropathy to healthy controls. For this analysis, only the healthy eyes were included. An overview of the participants of the initial umbrella study and the subgroup used for this study can be found in Figure 1.

Data for the healthy participants were initially randomly collected from healthy subjects during a routine clinical visit. Inclusion criteria for this group were age greater than 18 years and the absence of any ocular diseases. Written informed consent was obtained from all participants. The iris color data were included after an assessment by an expert ophthalmologist and categorized into the predominant and generally comprehensible colors of brown, green, and blue. This study involving human participants was performed in accordance with the tenets of the Declaration of Helsinki and was approved by the Ethics Committee of Northwest and Central Switzerland (EKNZ TIBE-15/90).

### Dynamic OCT Pupillometry

All data were acquired with dynamic OCT pupillometry using the swept-source DRI OCT-1 Atlantis (Topcon, Tokyo, Japan) with software version 9.12.003.04.^4^ To acquire 3D pupillary volumes, the OCT signal was first focused on the corneal epithelium, and the scan area was then shifted posteriorly until the pupil area was centered on the display of the OCT device. Each volume consisted of 256 B-scans with 512 × 992 pixels, covering 6.0 mm × 6.0 mm × 2.6 mm. The smallest possible fixation target was used to minimize the perceived illumination from the device. No fundus photographs were performed, and no eye drops affecting the pupil size were administered. Participants were instructed to remain in a relaxed position and to mention any discomfort that could alter the autonomic nervous system.

Two measurements per eye were performed, one in scotopic and the other in photopic light levels. The scotopic light conditions were achieved by darkening the room and covering the head of the patient with a light protecting drape frequently used for eye examinations (Oculus, Wetzlar, Germany). The photopic conditions were generated with a daylight therapy lamp (TL 90; Beurer GmbH, Ulm, Germany) providing a flicker-free and ultraviolet-free light. This daylight lamp emitted a light intensity of approximately 10,000 lux at a 15-cm distance with a color temperature of 6500K. The measurements in photopic conditions were made at least 1 minute after the scotopic measurements to allow the pupil to adapt.

### Data Processing and Analysis

**Figure 2.**
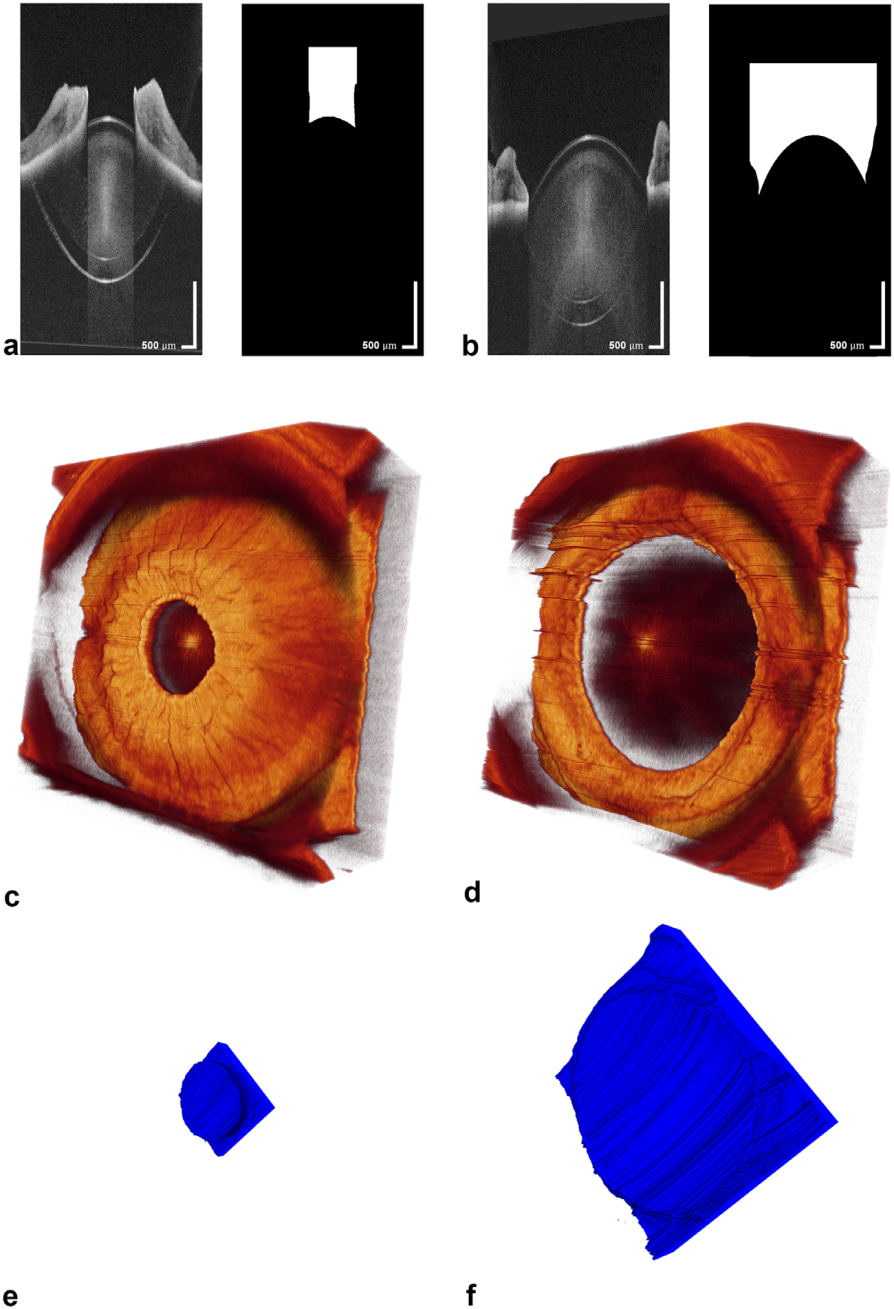
Image processing for dynamic volume-rendered OCT pupillometry. (a, b) B-scans in miosis (a) and mydriasis (b) with the corresponding IPS segmentations. (c, d) Volume-rendered OCT acquisitions in photopic (c) and scotopic (d) conditions, anterior visualization. (e, f) Corresponding volume-rendered IPS segmentations, posterior visualization. The volume rendering visualizes the structural differences between the photopic (e) and scotopic (f) states and corresponds to the differences in aqueous humor inside the IPS.

Image data were exported from the OCT device in bitmap (.bmp) format and freed from speckle noise by applying image postprocessing with structural guided filtering.^20^ The files were imported as an image sequence into ImageJ 1.48b (National Institutes of Health, Bethesda, MD), where the 3D IPS was delimitated with a bounding box.^21^ For generalizability, the lateral IPS borders were identified in an en face OCT projection, and the depth of the IPS was determined with a fixed axial offset from the lens apex of 200 pixels.^4^ Within the cropped bounding box, the IPS was further annotated pixel-wise and binarized. These generated IPS volumes were imported into Amira 5.6.0 (Thermo Fisher Scientific, Waltham, MA). The image processing for dynamic volume-rendered OCT pupillometry is represented in Figure 2. Calculations for the volume and surface area were performed with the real voxel sizes based on a triangular surface grid.

These values were used as the baseline values for the scotopic and photopic volumes (mm^3^). Additionally, further functional parameters, such as the pupillary ejection fraction (PEF), 3D contractility, and relative light response (RLR), were calculated as described previously.^4^ To analyze the ejection fraction of aqueous humor analogous to the left ventricular ejection in the heart, PEF was calculated as

$$\begin{array}{ccc} & & \;Pupillary\;ejection\;fraction\;\left[\%\right]\\ & & \;\;\;\;=\frac{Scotopic\;volume\;\left[mm^{3}\right]-Photopic\;volume\;\left[mm^{3}\right]\;}{Scotopic\;volume\;[mm^{3}]}\times 100\; \end{array}$$

Volumetric measurements enable a quantification of the difference in the amount of aqueous humor between the scotopic and photopic state. For a measurement of the maximal volume change between the states (corresponding to the amount of aqueous humor that can be pumped out with a maximal contraction), the 3D contractility was calculated as follows:

$$\begin{array}{ccc} & & 3D\;contractility\;\left[mm^{3}\right]\\ & & \;=Scotopic\;volume\;\left[mm^{3}\right]-Photopic\;volume\;\left[mm^{3}\right]\; \end{array}$$

Because the absolute volume change can remain similar between different participants with a variant ratio, the ratio between the scotopic and photopic volume was calculated as RLR. The formula indicates how much more aqueous humor is present in the scotopic state compared to the photopic state:

$$\begin{array}{c} Relative\;light\;response\;\left[\mathrm{ratio}\right]=\frac{Scotopic\;volume\;\left[mm^{3}\right]\;}{Photopic\;volume\;\left[mm^{3}\right]}\; \end{array}$$

### Statistical Analysis

The statistical analysis was performed with R 4.0.3 (R Foundation for Statistical Computing, Vienna, Austria) and Python 3.8 (Python Software Foundation, Wilmington, DE). A correlation analysis was performed on the volumetric measurements and age to investigate linear correlations. Analysis of variance (ANOVA) models were fit to the data with type II estimation of sum of squares. Independent variables were iris color (categorical), sex (categorical), and age (continuous). Dependent variables were the scotopic volume, the photopic volume, PEF, 3D contractility, and RLR. Statistical hypothesis tests were performed to detect significant effects of iris color, age, and sex on each of the volumetric measurements. Cook's distance was analyzed to check for influential data points. Additional Pearson correlation analyses were performed to determine associations between the contractility parameters and intraocular pressure, as well as between the contractility parameters and the spherical equivalent of the eye.

The data in this work are presented as mean ± standard deviation (SD). Statistical significance in this work was calculated for α = 0.05. To show gradual differences at the significance level, the following labeling is used in the figures and the table: not significant (n.s.) ≙*P* ≥ 0.05, *≙0.05 > *P* ≥ 0.01, **≙0.01 > *P* ≥ 0.001, ***≙0.001 > *P* ≥ 0.0001, ****≙*P* < 0.0001. The results were visualized with R 4.0.3 and Python 3.8. Reproducibility of the method has been previously presented with an intragrader coefficient of variation ranging from 0.58% to 2.17%, accounting for 79.2% of the total variability, whereas the intergrader variability accounted for 20.8% of the total variability.^4^

## Results

The study population consisted of 40 study participants, for which 71 IPSs were analyzed. IPS data were available for 45 female and 26 male eyes. The mean age of the population was 51.1 ± 16.6 years (range, 20‒77). Compared to the initial umbrella study, one additional IPS was excluded due to imaging artifacts, so data from a total of nine eyes were of insufficient quality for the analysis (Fig. 1). The presented dataset consisted of 30 IPSs from brown irises, 10 IPSs from green irises, and 31 IPSs from blue irises. The mean spherical equivalent was 0.1 ± 2.0 diopters (range, −6.375 to 3.250), and the mean intraocular pressure was 14.5 ± 2.0 mm Hg (range, 11‒19).

### Association Among Outcomes

**Figure 3.**
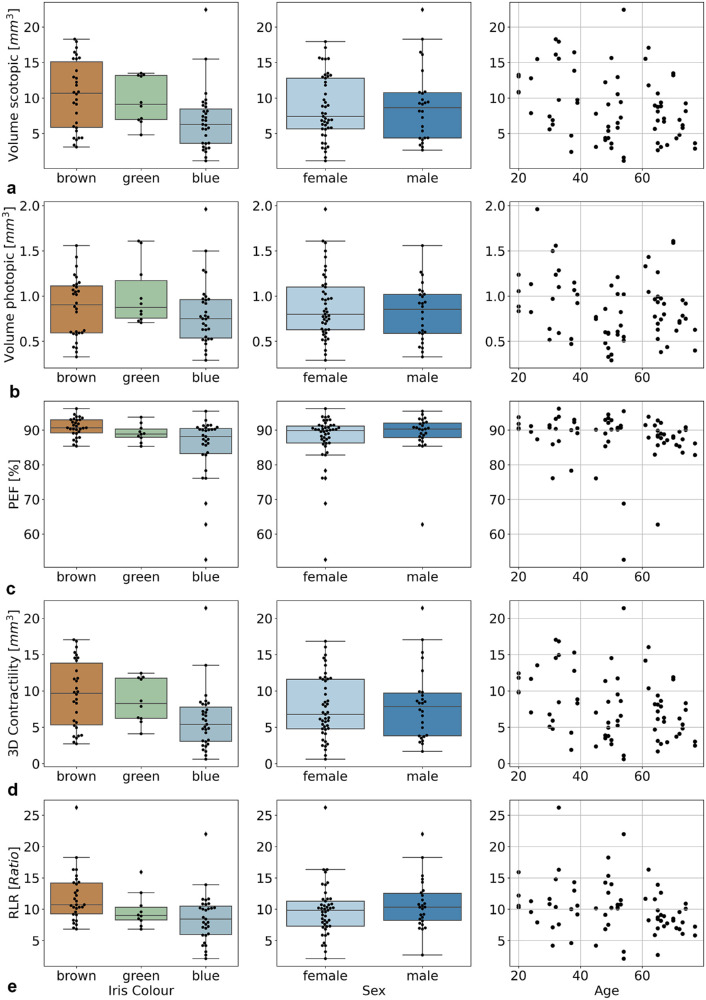
Summary statistics of pupillary contractility parameters with regard to iris color, sex, and age. The three analyzed eye colors and sex are presented as boxplots with medians and interquartile ranges, and the influence of age is shown as scatterplots. (a) Scotopic volume corresponds to the volume measured in a darkened room. (b) Photopic volume corresponds to the volume under lighting conditions. (c–e) Functional contractility parameters are described for PEF (c), 3D contractility (d), and RLR (e).

**Figure 4.**
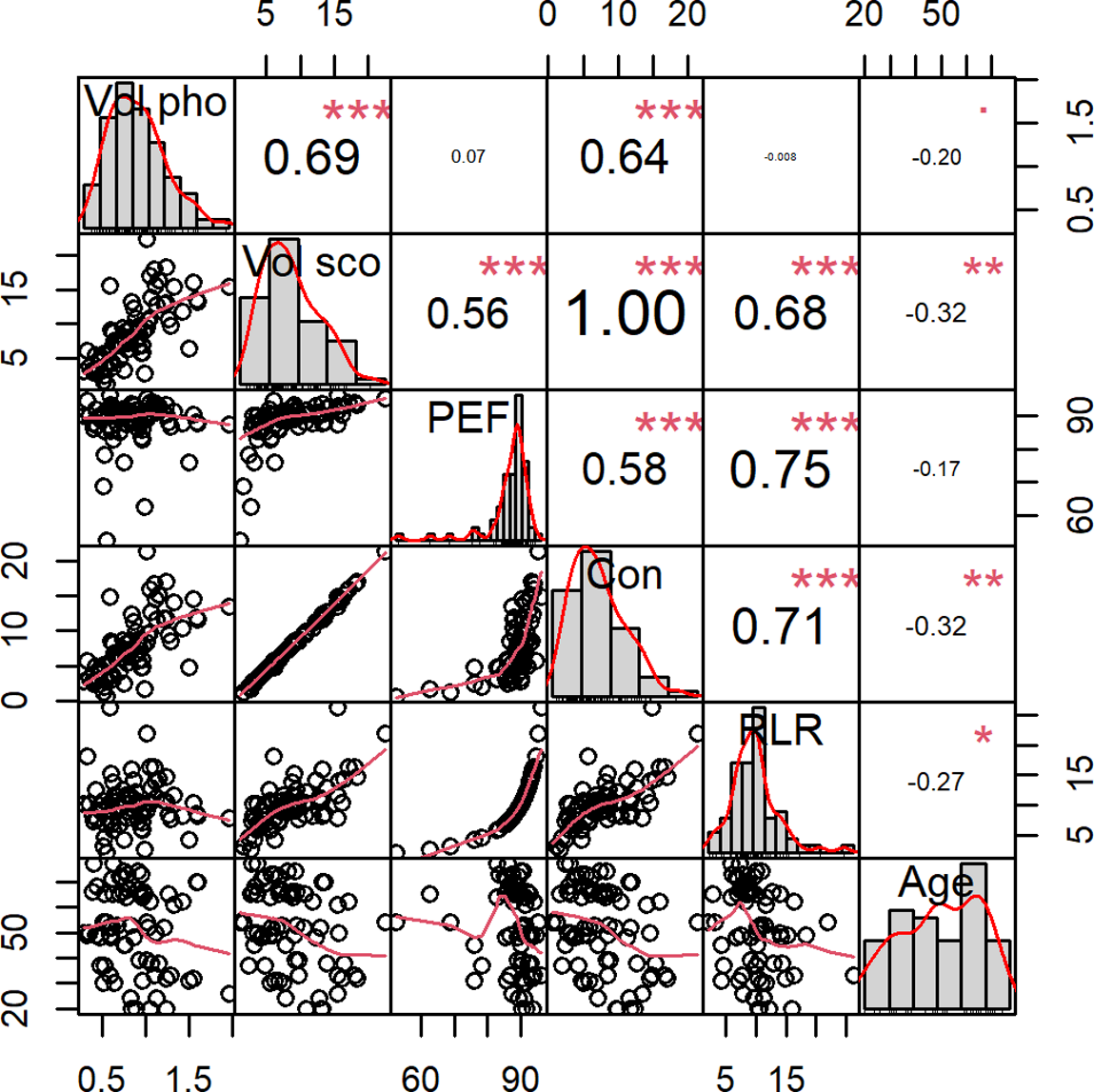
Correlation matrix chart for the variables Vol pho, Vol sco, PEF, Con, RLR, and age. Plots on the matrix diagonal show histograms with density estimators in *red*. Numbers in the upper half of the matrix are Pearson correlation coefficients, with asterisks indicating results of tests of no correlation based on Pearson's product moment correlation coefficient. Plots in the lower half of the matrix are bivariate scatterplots with fitted lines in *red*. Vol pho, photopic volume; Vol sco, scotopic volume; Con, 3D contractility.

The correlation analysis revealed that the volumetric parameters were linearly correlated. The highest linear correlation was found between the baseline parameter scotopic volume and the 3D contractility. As the participants in this study population had a functioning pupil, the pupil could contract to a very small volume in the photopic state. This photopic volume was almost marginal when subtracted from the scotopic volume (Fig. 3). Hence, the functional measurements were strongly correlated with the baseline measurements, as the contractility analysis was correlated with the extreme values of photopic and scotopic IPS (Fig. 4). An overview of the *P* values of the ANOVA analyses and *t*-tests between different iris color evaluation parameters is provided in the Table.

**Table.** ANOVA Results Showing the Effects of Iris Color, Age, and Sex on Scotopic Volume, Photopic Volume, PEF, 3D Contractility, and RLR

	*P*		
Parameter	Color	Age	Sex
Scotopic volume (mm^3^)	0.01804*	0.02242*	0.94136
Photopic volume (mm^3^)	0.21690	0.07152	0.70022
PEF (%)	0.01039*	0.39979	0.68538
3D contractility (mm^3^)	0.01554*	0.02380*	0.96366
RLR (ratio)	0.02575*	0.10782	0.81554

PEF = pupillary ejection fraction, RLR = relative light response, * = statistically significant result

### Baseline Measurements

In all participants, the scotopic IPS was at least 2.11 times larger than the photopic IPS, demonstrating that the light adaptation worked. The ANOVA analyses revealed that the scotopic IPS was influenced by iris color, with the brown irises being the widest (brown, 10.39 ± 4.86 mm^3^; green, 9.68 ± 3.31 mm^3^; blue, 6.75 ± 4.27 mm^3^; *P* = 0.018). The scotopic IPS decreased significantly with increasing age (*P* = 0.022). Photopic IPS was not influenced by iris color, age, or sex. The baseline measurement results are visualized in Figure 3.

### Functional Measurements

Iris color was significantly correlated with PEF (*P* = 0.010). The pupils of brown irises had a higher PEF (90.8% ± 2.7%) than green irises (89.1% ± 2.5%) and blue irises (85.0% ± 9.3%), and PEF decreased gradually with a decrease in pigmentation. Age and sex did not influence PEF. A visual representation of the functional measurements is shown in Figure 3.

 The 3D contractility was greater in more pigmented irises (brown, 9.52 ± 4.59 mm^3^; green, 8.66 ± 3.07 mm^3^; blue, 6.44 ± 4.87 mm^3^; *P* = 0.016). The 3D contractility significantly decreased with age (*P* = 0.024) but did not differ between men and women (*P* = 0.964). The gradual decrease in contraction function with lower iris pigmentation was also present in the RLR (brown, 11.90 ± 4.03; green, 9.75 ± 2.73; blue, 8.52 ± 3.88; *P* = 0.026). Older participants showed a non-significant tendency for a reduced RLR. Sex had no significant effect on the RLR (*P* = 0.816).

When analyzing Cook's distance of the multiple linear regression models obtained from the ANOVA analyses, no influential data points were found. Further, the correlation analyses between intraocular pressure and the functional measurements showed no significant Pearson correlation (PEF: *r* = 0.08, *P* = 0.54; 3D contractility: *r* = −0.02, *P* = 0.89; RLR: *r* = 0.08, *P* = 0.54). There was no significant Pearson correlation between the spherical equivalent and the functional measurements (PEF: *r* = 0.03, *P* = 0.80; 3D contractility: *r* = 0.12, *P* = 0.35; RLR: *r* = 0.07, *P* = 0.58).

## Discussion

This study retrospectively assessed the natural variability in pupillary contractility using a commercially available OCT device in healthy Caucasian participants. The pupillary contractility was assessed with dynamic volume-rendered OCT pupillometry by comparing the pupil size in scotopic and photopic light conditions. The main finding was that pupillary contractility parameters were significantly affected by iris color (with more pigmented irises having greater contractility) and age (with contractility decreasing with increasing age). These results indicate that iris color and age should be considered when comparing results from pupillary contractility testing.

Brown irises contain more melanin and are more pigmented than green irises and blue irises.^22^ To date, few studies have investigated the effect of iris color on pupillary contractility, and they have shown contradictory results.^17^^,^^19^^,^^23^ The present dynamic OCT pupillometry method quantifies the pupil volume in 3D on a micrometer scale compared to clinically used two-dimensional analyses. The difference in contractility based on iris color was true for all three evaluated contractility parameters: PEF, 3D contractility, and RLR. In the literature, a higher contractility for brown eyes compared to blue eyes was also found in a previous study, with the contraction amplitude being higher for the brown irises.^17^ In contrast to our study, a previous study by Bergamin et al.^17^ investigated short-term contractions without measurements in miotic and mydriatic conditions. To further investigate reasons for differences among iris colors, the biomechanical tissue properties could be measured with techniques such as optical coherence elastography in upcoming studies.^24^

In contrast to hair or skin pigmentation, iris color is often disregarded in clinical practice in large research databases such as the UK Biobank,^25^ which is surprising because of the known clinical differences in terms of iris color. Drugs affecting pupil size have been shown to have different effects on irises with different colors. For example, 1% tropicamide showed a stronger increase in pupil diameter and shorter time to maximal effect for bluer irises in iris photography.^26^ Further, iris pigmentation is a known risk factor for uveal melanoma, as people with a blue iris color have a higher risk of being affected than those with brown eyes.^27^ In general, gathering information on iris color in future studies would be necessary to analyze its effects on further outcome parameters. A remaining challenge is a unified classification of iris colors. A widely accepted classification system is lacking, and various groupings of iris color have been presented.^28^

The second main finding of our study is that pupillary contractility decreases with increasing age. This can be mostly explained through reduced scotopic volume, which leads to a reduced overall change in volume. This reduced scotopic volume in older participants was described as early as the 1950s and confirmed in subsequent studies.^19^^,^^29^ It is noteworthy that the other contractility parameters in our study—PEF and RLR—did not show a significant change with age. These two parameters describe relative changes in pupil size, which are not as strongly correlated with scotopic volume as pupillary contractility. As the scotopic volume is mainly generated through the activity of the iris dilator muscle, a specific investigation of its activity with age could provide further insight into the mechanisms of decreased pupillary contractility.

We found no significant effect of sex on any of the contractility parameters. The long-held belief that women have a higher contractility than men was shown to be incorrect in a previous study.^19^ Our study confirms this finding of no differences. Further, the intraocular pressure and the spherical equivalent were not correlated with the contractility parameters. Hence, the three parameters of sex, intraocular pressure, and spherical equivalent do not seem to affect pupillary contractility.

This study was limited by the relatively low number of participants with green irises (*n* = 10) which led to an unequal distribution among the color groups; however, this was due to the retrospective study design, as iris color was not a parameter of the inclusion criteria in the initial umbrella study.^4^ The iris color distribution of the present study corresponds relatively well to the variability found in Caucasian populations.^30^ Furthermore, the overall presented results indicate an influence of iris color at a group level, with brown eyes being more contractile than blue eyes. However, the largest contractility of a single eye was shown in a blue eye, and there was a large overlap of values among the groups. Previous results have been inconclusive regarding the effect of iris color on pupillary contractility, with some studies showing a larger contractility in brown irises, a larger baseline pupil size in blue irises, or no effect of iris color on pupillary contractility.^17^^–^^19^ A further limitation of the current study was the categorization of the iris color by a single expert into the predominant colors; however, due to the nature of the retrospective analysis, a generally comprehensible method for iris classification was used. In upcoming studies, standardized assessments could be used to better differentiate color nuances.^31^ Further parameters beyond iris color, such as genetic expression, body composition, light sources used, and behavioral factors, could be included in future work to find interdependencies between different factors potentially affecting contractility. Further, the use of multiple ANOVA analyses despite linear correlations between the dependent variables might also be considered a limitation; however, these correlations were due to the healthy state of the participants. When investigating disease states impacting contractility, such as non-arteritic anterior ischemic optic neuropathy, the correlations between the baseline and functional parameters are not the same, and significant differences exist in the baseline values.^4^ Hence, we performed ANOVA analyses for all evaluation parameters to show the impact of iris color, age, and sex in a healthy population as a reference.

With our work, we propose a volumetric approach to analyzing the naturally occurring variability in pupillary contractility. Compared to previous two-dimensional analyses, dynamic OCT pupillometry provides a precise volumetric analysis on a micrometer scale and a quantification of the aqueous humor in the IPS. Hence, this natural variability analysis can provide a baseline comparison for volumetric pupillary contractility measurements and aqueous humor quantification in further studies.

## Conclusions

The natural variability in pupillary contractility can be analyzed with dynamic OCT pupillometry using a commercially available OCT device. With this method, iris color and age were shown to have a significant effect on the maximal pupillary contractility and the IPS in scotopic conditions. This study suggests that iris color and age should be considered factors impacting pupillary response.
